# Survival prediction of tuberous sclerosis complex gene variant in patients with advanced non-small-cell lung cancer treated with platinum doublet

**DOI:** 10.1042/BSR20181426

**Published:** 2019-03-19

**Authors:** Jeong-Seon Ryu, Jun Hyeok Lim, Hyun-Jung Kim, Min Jeong Kim, Mi Hwa Park, Jung Soo Kim

**Affiliations:** Department of Internal Medicine, Inha University Hospital, Incheon, Korea

**Keywords:** cisplatin, survival, tuberous sclerosis complex, validation

## Abstract

Tuberous sclerosis complex (TSC) 1 and 2 function as tumor suppressors by inactivating the mammalian target of rapamycin (mTOR) pathway. Although the effect of platinum on TSC function has been studied, associations between *TSC* gene variants and survival of cancer patients treated with platinum-based chemotherapy were not evaluated. Genetic variants of *TSC1* and *TSC2* were identified by next-generation sequencing and selected for further clinical evaluation based on predetermined criteria. Associations of the gene variants with treatment outcomes (progression-free survival, PFS; overall survival, OS) were evaluated in testing and validation sets of patients with advanced non-small-cell lung cancer (NSCLC). Hazard ratios (HRs) and 95% confidence intervals (CIs) were estimated with the multivariable Cox model. The *TSC1* Met322Thr (rs1073123) variant met the criteria for further analysis in testing and validation sets each containing 183 patients. The median PFS for the 366 patients was 4.9 months. Fifty-three patients (14.5%) had the *TSC1* (Met322Thr or Thr322Thr) variant. *TSC1* Met322Thr associated with longer PFS in the testing set (HR adjusted for age, gender, smoking habits, Eastern Cooperative Oncology Group performance status, histology, and stage [aHR] and 95% CI: 0.63 and 0.45–0.87, Cox *P*=0.009), and this was confirmed in the validation set (aHR and 95% CI: 0.58 and 0.36–0.93, Cox *P*=0.004). However, no association was found between the *TSC1* gene variant and OS. These findings suggest that the *TSC1* gene variant is an important predictive marker for platinum doublet chemotherapy outcomes in NSCLC patients.

## Introduction

Lung cancer is the most common cause of cancer-related mortality worldwide. The 5-year survival of patients with advanced stage non-small-cell lung cancer (NSCLC) remains disappointingly low at 16%. Despite recent advances in targeted immunological therapies, platinum doublet chemotherapy still plays a pivotal role in the treatment of advanced NSCLC.

It is not fully understood how cisplatin acts on cancer cells, but accumulating evidence indicates that cisplatin forms DNA adducts; thereby, inhibiting replication and transcription, which results in cell cycle arrest and apoptosis. Therefore, the development of biomarkers to predict the effects of platinum on NSCLC has focused on DNA damage responses, including homologous recombination, nucleotide excision repair, and mismatch repair [1]. In addition, the phosphoinositide-3-kinase catalytic subunit-α (PI3KCA)/AKT/mammalian target of rapamycin (mTOR) signaling pathway has also been investigated to identify platinum resistance mechanisms and to predict platinum effects [2–5]; however, the clinical relevance of this pathway in NSCLC has not yet been validated. Further, pyruvate dehydrogenase kinase (PDK) and AMP-activated protein kinase activities were enhanced in stomach, liver, and colon cancer cell lines that were treated with cisplatin, and these two kinases contributed to cell survival via interactions with tuberous sclerosis complex (TSC) 1 and TSC 2 [6–8].

The *TSC1* and *TSC2* genes are located at chromosomes 9q34 and 16p13.3, respectively, and according to Knudson’s tumor suppressor model, it has been established that *TSC1* and *TSC2* are involved in the development of TSC syndrome [9]. *TSC1* and *TSC2* encode for hamartin and tuberin, respectively. The hamartin and tuberin heterodimer has been shown to function as a tumor suppressor by inactivating mTOR through suppression of the small GTPase Rheb (Ras-homolog enriched in brain). However, the clinical implications of genetic variations in *TSC1* or *TSC2* in cancer patients have not yet been elucidated.

In this study, we screened for genetic variants of *TSC1* and *TSC2* and associated genes to determine whether genetic variants associated with platinum doublet chemotherapy outcomes in NSCLC patients.

## Methods

### Selection of study population and acquisition of clinical information

From over 500 NSCLC patients with stage III or IV disease who were diagnosed between March 2000 and December 2005 as part of the Lung Cancer Cohort of Inha University Hospital (Incheon, South Korea) [10], we selected 368 patients who were treated with more than two cycles of platinum-based chemotherapy as a first-line treatment (Supplementary Figure S1). Patients who were evaluated after every two or three chemotherapy cycles, who had complete follow-ups at Inha University Hospital, and whose peripheral blood lymphocytes were available for analysis were included in this study. Information regarding treatment, tumor response, follow-up, survival, smoking habits, and performance status according to the Eastern Cooperative Oncology Group (ECOG) were collected. The patients’ clinical stages were reassessed according to the 7th edition of the Tumor Node Metastasis classification system [11]. Patient response to platinum doublet treatment, which is a secondary endpoint, was updated according to the Response Evaluation Criteria in Solid Tumors (RECIST) version 1.1 [12]. A total of 366 patients were randomly assigned to two groups for testing and validation using the Zelen permuted block randomization method [13]. This study was approved by the Institutional Review Board of Inha University Hospital.

### Selection of genetic variants of candidate genes and genetic analysis of TSC1

DNA was isolated from the buffy coat and quality control was performed (Supplementary Method). Next-generation sequencing was performed on an Illumina Hiseq2000 platform, and a custom panel composed of 150 cancer-related genes was used for the initial screening of 24 patients with advanced stage NSCLC. Among the 150 genes, *TSC1, TSC2*, and the related genes *PI3KCA, AKT1, mTOR*, and *PDK1* were selected for this study (Supplementary Table S1). Thirty-three genetic variants of *TSC1*, 22 variants of *TSC2*, 9 variants of *PI3KCA*, 44 variants of *AKT1*, 52 variants of *mTOR*, and 21 variants of *PDK1* were identified. A genetic variant was determined to have a clinical association if it met the following criteria: minor allele frequency >5%, call rate >90%, Hardy–Weinberg equilibrium *P*-value >0.001, and a high or moderate effect impact according to the SnpEff variant prediction program [14]. The *TSC1* Met322Thr (rs1073123) variant met the criteria and was chosen for further analysis. Genotyping for the *TSC1* Met322Thr variant was performed using the TaqMan assay (Applied Biosystems).

### Clinical endpoint analysis

The primary endpoint in this study was progression-free survival (PFS) from the start date of chemotherapy to recurrence. Patients who were still alive and progression-free at the end of the follow-up were treated as censored at the date of follow-up. The secondary endpoint was overall survival (OS), which was calculated from the time of diagnosis to the time of the last follow-up or death due to any cause.

### Statistical analysis

The characteristics of the two groups within the study population were compared using the χ^²^ test. The effect of an individual clinical variable or genetic variant of *TSC1* on survival was estimated using the Kaplan–Meier method and log-rank testing. Observations were censored at survival, loss to follow-up or death from other causes. The hazard ratios (HRs) and 95% confidence intervals (CIs) for all of the clinical variables were estimated using the Cox proportional hazards model. Significance was determined using a two-tailed test and *P*-values <0.05 were considered significant. Analyses were performed using the IBM SPSS statistical software package (version 19.0; SPSS Inc.; Chicago, IL, U.S.A.) and Stata (version 12.1; StataCorp, Ltd.; College Station, TX, U.S.A.).

## Results

### Patient characteristics

The clinical variables and the *TSC1* variants for all of the patients in the cohort are shown in Table 1. For the first-line regimen, the gemcitabine doublet was given to 124 patients (34%) and the taxane doublet was given to 112 patients (31%). Three hundred and thirteen of the patients (85%) had the wild-type *TSC1* genotype and 53 patients (14.5%) had a variant *TSC1* genotype (52 had the Met322Thr variant and two had the Thr322Thr variant). Progression after chemotherapy was observed in 344 patients (94%). There were no differences in the clinical variables, responses to chemotherapy, and *TSC1* gene variants between the testing and validation sets.

**Table 1:** Clinical characteristics of the patients in testing, validation, and combined sets

		Combined, %		Testing, %		Validation, %		χ^²^ *P*
Age	Median (min–max)	65.0 (32–86)		63.5 (34–80)		65.1 (32–86)		0.375
Gender	Women	101	27.6	57	56.4	44	43.6	0.168
	Men	265	72.4	126	47.5	139	52.5	
Smoking habit	Never	102	27.9	55	53.9	47	46.1	0.352
	Ever	263	72.1	127	48.3	136	51.7	
Histology	ADC	200	54.6	101	50.5	99	49.5	0.943
	SQC	133	36.3	65	48.9	68	51.1	
	Others	33	9.0	17	51.5	16	48.5	
ECOG PS	0–1	298	82.1	151	50.7	147	49.3	0.413
	2 or more	65	17.9	29	44.6	36	55.4	
Stages	IIIA	36	9.8	17	47.2	19	52.8	0.923
	IIIB	91	24.9	45	49.5	46	50.5	
	IV	239	65.3	121	50.6	118	49.4	
First-line regimens	Platinum plus							
	Gemcitabine	124	33.9	62	50.0	62	50.0	0.972
	Taxane	112	30.6	58	51.8	54	48.2	
	Irinotecan	95	26.0	45	47.4	50	52.6	
	Pemetrexed	26	7.1	13	50.0	13	50.0	
	Others	9	2.4	5	55.6	4	44.4	
No. cycles	2	92	25.2	46	50.0	46	50.0	0.769
	3–4	159	43.6	81	50.9	78	49.1	
	4–6	113	31.0	55	48.7	58	51.3	
	7	1	0.2	0	0.0	1	100.0	
Response	CR or PR	146	39.9	66	45.2	80	54.8	0.252
	SD	97	26.5	55	56.7	42	43.3	
	PD	122	33.3	61	50.0	61	50.0	
	Not evaluated	1	0.2	1	100.0	0	0.0	
*TSC1* Met322Thr	Met/Met	313	85.5	156	49.8	157	50.2	0.989
	Met/Thr	51	14.0	26	51.0	25	49.0	
	Thr/Thr	2	0.5	1	50.0	1	50.0	
PFS	Median, months (95% CIs)	4.9 (4.57–5.29)		4.7 (4.21–5.18)		5.1 (4.47–5.59)		0.272^a^
	Event	344		176		168		

*P*-values indicates χ^²^ testing between testing and validation except ^a^log-rank test.

Abbreviations: ADC, adenocarcinoma; CR, complete remission; ECOG PS, ECOG performance status; PD, progressive disease; PFS, progression free survival; PR, partial remission; SD, stable disease; SQC, squamous cell carcinoma.

### Effect of the TSC1 gene variant on survival of patients in the testing set

**Figure 1:**
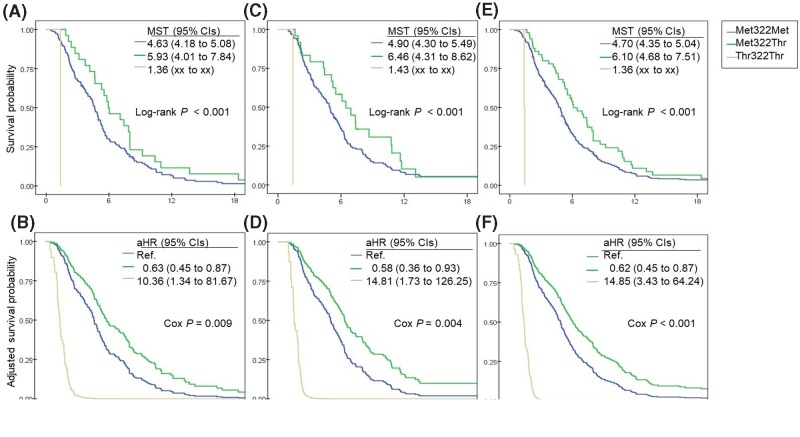
PFS of patients by the *TSC1* genetic variant PFS of patients by *TSC1* genetic variant in the testing (**A,B**), validation (**C,D**), and combined (**E,F**) sets by Kaplan–Meier plot and the Cox proportional hazard model.

Histology and response to platinum doublet associated with PFS (log-rank *P*=0.001 and <0.001, respectively). Disease stage also associated with PFS (log-rank *P*=0.056). The median PFS for patients with the *TSC1* Met322Thr variant was 5.9 months and was longer than the median PFS for patients with the *TSC1* Met322Met variant (log-rank *P*<0.001; Figure 1 and Supplementary Table S2). After adjusting for confounding variables, including age, gender, ECOG performance status, smoking habits, histology, and disease stage, the Cox model showed that patients with the *TSC1* Met322Thr variant had longer PFS than patients with the *TSC1* Met322Met variant (HR adjusted for age, gender, smoking habits, ECOG performance status, histology, and stage [aHR] and 95% CI: 0.63 and 0.45–0.87, Cox *P*=0.009). However, the *TSC1* gene variants did not affect OS (median survival time [MST] in months and 95% CIs: 14.1 and 10.8–17.4 for Met322Met; 16.4 and 11.1–21.8 for Met322Thr; log-rank *P*=0.461; Supplementary Figure S2).

### Effect of the TSC1 gene variant on survival of patients in the validation set

Gender, histology, stage, first-line regimen, and the response to platinum doublet associated with PFS (log-rank *P*=0.031, 0.004, <0.001, 0.002, and <0.001, respectively). The median PFS for patients with the *TSC1* Met322Thr variant was 6.4 months and was longer than the median PFS for patients with the *TSC1* Met322Met variant (log-rank *P*<0.001). After adjusting for confounding variables, the Cox model showed that patients with the *TSC1* Met322Thr variant had longer PFS than patients with the *TSC1* Met322Met variant (aHR and 95% CI: 0.58 and 0.36–0.93, Cox *P*=0.004). However, the *TSC1* gene variants did not affect OS (MST, months and 95% CIs: 13.8 and 11.4–16.1 for Met322Met; 17.1 and 14.7–19.5 for Met322Thr; log-rank *P*=0.641).

### Effect of the TSC1 gene variant on survival of patients in the combined set

When associations between clinical variables and PFS were analyzed in the entire cohort using the log-rank test, we found that smoking habits, histology, stage, and first-line regimen associated with PFS. The multivariate Cox proportional hazard model was performed to further assess the effects of these variables. Histology and stage affected PFS (Cox *P*=0.008 and 0.003, respectively). Age and gender associated marginally with shorter PFS (aHR and 95% CIs: 1.01 and 1.00–1.03 for age; 1.43 and 0.96–2.13 for gender) (Table 2). The median PFS for patients with the *TSC1* Met322Thr variant was 6.1 months and was longer than the median PFS for patients with the *TSC1* Met322Met variant (log-rank *P*<0.001). The Cox proportional hazard model also showed that patients with the *TSC1* Met322Thr variant had longer PFS than patients with the *TSC1* Met322Met variant (aHR and 95% CIs: 0.63 and 0.45–0.87, Cox *P*<0.001). However, the *TSC1* gene variants did not affect OS (MST, months and 95% CIs: 13.9 and 12.0–15.7 for Met322Met, 16.7 and 13.9–19.5 for Met322Thr; log-rank *P*=0.359).

**Table 2:** Effects of clinical variables or genetic variation of *TSC1* on PFS in combined set

		aHR	95% CI	Cox *P*
Age	Increasing	1.01	1.00–1.03	0.085
Gender	Women	Ref.		0.077
	Men	1.43	0.96–2.13	
ECOG PS	0–1	Ref.		0.930
	2 or more	1.01	0.75–1.36	
Smoking habit	Never	Ref.		0.278
	Ever	0.80	0.54–1.19	
Histology	ADC	Ref.		0.008
	SQC	0.66	0.50–0.85	
	Others	0.76	0.56–1.32	
Stages	IIIA	Ref.		0.003
	IIIB	1.49	0.95–2.32	
	IV	2.02	1.30–3.14	
*TSC1* Met322Thr	Met/Met	Ref.		<0.001
	Met/Thr	0.62	0.45–0.87	
	Thr/Thr	14.86	3.43–64.24	

Abbreviations: ADC, adenocarcinoma; ECOG PS, ECOG performance status; SQC, squamous cell carcinoma.

## Discussion

This study found that a genetic variant of the *TSC1* gene is a robust predictor of the effects of platinum doublet therapy in patients with advanced stage NSCLC. This finding supports the model in which platinum acts on mTOR signaling through TSC1. TSC1 is composed of 1164 amino acids and the region that is responsible for interaction with TSC2 is amino acid 302–430 [9], which is also the region where the TSC1 variants exist. Therefore, we suggest that the *TSC1* missense variant Met322Thr affects the stability of TSC2, which results in inhibition of mTOR and cellular growth and proliferation. Our data that patients with *TSC1* Met322Thr had longer PFS in the testing and validation sets support this hypothesis.

We have not provided evidence for the biological basis of the predictive value of the *TSC1* gene variant. However, previous experimental studies have demonstrated the effects of platinum on TSC1. It has been shown that susceptibility to cell death increased upon DNA damage by an alkylating agent in a *TSC1*-deficient cell line [15]. In addition, the mTOR survival pathway was activated in lung and ovarian cancer cell lines that were treated with cisplatin, and sensitivity to cisplatin was enhanced by inhibiting the mTOR pathway [2, 3]. Further, preclinical and clinical data has shown a synergistic effect with cisplatin and an mTOR inhibitor [16]. In summary, DNA-damaging agents, including cisplatin, can activate PDK1, which results in inhibition of the mTOR survival pathway through TSC1 [6].

The results of this study should be interpreted with some caution. First, PFS was evaluated as an endpoint, but the PFS measurements may be not precise due to evaluation or measurement bias in the retrospective study design [17]. Nevertheless, responses were evaluated after every two to three cycles of platinum doublet using criteria from RECIST version 1.1. Second, druggable mutations, including *EGFR* activating mutations, and their effects on survival were not evaluated in this study because more than half of the patients were included before this testing was available in Korea. In the testing, validation, and combined data sets, the *TSC1* gene variant did not affect the use of targeted agents post-platinum doublet chemotherapy (data not shown). Regardless of these limitations, we believe that this study contributes translationally relevant information on *TSC1* gene variants. In conclusion, we found that the *TSC1* Met322Thr variant plays a predictive role in NSCLC patients treated with platinum doublet.

## Perspectives

- Platinum doublet chemotherapy still play a pivotal role in treatment of advanced NSCLC, its predictive biomarker remains unknown.
- Genes related to mTOR pathway were analyzed with next-generation sequencing. We found that TSC1 Met322Thr conferred longer PFS in both testing and validation set (aHR and 95% CIs: 0.63 and 0.45–0.87, Cox *P*<0.001).
- TSC1 gene variant is an important predictive marker for platinum-based chemotherapy outcomes in NSCLC patients.

## Supporting information

**Supplementary Fig S1:** Patients’ enrollment and study scheme

**Supplementary Fig S2:** Overall survival of the patients by genetic variation of *TSC1* gene in testing (A), validation (B), and combined (C) sets: Kaplan-Meier plot

**Supplemental Table S1:** Information on genetic variations of *TSC1* or *TSC2* gene and their related genes identified by next generation sequencing

**Supplemental Table S2:** Progression-free survival of the patients by clinical characteristics and genetic variation of of *TSC1* gene in testing, validation, and combined sets

## Abbreviations

aHR: HR adjusted for age, gender, smoking habits, ECOG performance status, histology, and stage
AKT: protein kinase B
AMP: adenosine monophosphate
CI: confidence interval
ECOG: Eastern Cooperative Oncology Group
EGFR: epidermal growth factor receptor
HR: hazard ratio
mTOR: mammalian target of rapamycin
NSCLC: non-small-cell lung cancer
OS: overall survival
PDK: pyruvate dehydrogenase kinase
PFS: progression-free survival
PI3KCA: phosphoinositide-3-kinase catalytic subunit-α
RECIST: Response Evaluation Criteria in Solid Tumor
TSC: tuberous sclerosis complex
