# Supplementary material for: Survival prediction of tuberous sclerosis complex gene variant in patients with advanced non-small-cell lung cancer treated with platinum doublet
Source: Biosci Rep. 2019 Mar 19;39(3):BSR20181426. doi: 10.1042/BSR20181426 (PMC6422885; doi:10.1042/BSR20181426)
Supplement: Supplementary file 1 [file bsr-39-bsr20181426_Supp1.pdf]

Supplementary Information

Content:

Methods Targeted sequencing

DNA preparation and quality control

Genotyping Using the TaqMan assay

Targeted next-generation sequencing

Target regions were captured from 1 μg of genomic DNA using the Agilent SureSelect Custom kit following the manufacturer's protocols (Agilent, Santa Clara, CA, USA). Briefly, DNA was sheared using the Covaris system (Covaris, Woburn, MA, USA) and purified using Agencourt AMPure XP beads (Beckman Coulter, Brea, CA, USA). The ends of the fragments were repaired and adaptors were ligated to the fragments. The resulting DNA library was purified using Agencourt AMPure XP beads and amplified by PCR. The quality and quantity of the DNA library was assessed with the Agilent 2100 Bioanalyzer. The DNA library was captured by hybridization to biotinylated RNA library baits. Bound genomic DNA was purified with streptavidin-coated magnetic Dynabeads (Invitrogen, Carlsbad, CA, USA) and then re-amplified. The targeted DNA library was sequenced on an Illumina Hiseq2000 with 100 base pair paired-end reads using protocols recommended by the manufacturer (Illumina, San Diego, CA, USA).

DNA preparation and quality control

DNA was isolated from buffy coat with QuickGene mini-310 (KURABO Industries, Osaka, Japan) and QuickGene DNA whole blood kit S (KURABO Industries, Osaka, Japan). The quantity and quality of the samples were determined using the Nano-drop® 1000 spectrophotometer (Thermo Scientific, Waltham, MA, USA) and by gel electrophoresis using 1% Agarose E-gels (Invitrogen, Carlsbad, CA, USA).

The requirement for the genomic DNA source for the BioMark platform (Fluidigm) is high purity without degradation. DNA purity is indicated by OD_{260}/OD_{280} and OD_{260}/OD_{230} ratios. The OD_{260}/OD_{280} ratio should be 1.8–2.0, and the OD_{260}/OD_{230} ratio should be > 1.5. The BioMark platform requires a DNA concentration of 60 ng/μl.

Genotyping Using the TaqMan assay

The TaqMan assay (Applied Biosystems) was performed according to the manufacturer's instructions. Genotyping of *TSC1* and *TSC2* was performed using the TaqMan fluorogenic 5' nuclease assay (ABI, Foster City, CA, USA). The final polymerase chain reaction (PCR) volume was 5 μl and contained 10 ng of genomic DNA, 2.5 μl of TaqMan Universal PCR Master Mix, and 0.13 μl of 40X Assay Mix. The thermal cycle conditions were 50°C for 2 min to activate the uracil N-glycosylase and to prevent carry-over contamination, 95°C for 10 min to activate the DNA polymerase, and then 45 cycles of 95°C for 15 s and 60°C for 1 min. All of the reactions were performed using 384-well plates and a Dual 384-Well GeneAmp PCR System 9700 (ABI, Foster City, CA, USA). The endpoint fluorescent readings were

measured with an ABI PRISM 7900 HT Sequence Detection System (ABI, Foster City, CA, USA). Duplicate samples and negative controls were included to ensure the accuracy of the genotyping.

Supplementary Information

Content:

Figure S1 Patient enrollment and study scheme**.** ILCC, Inha Lung Cancer Cohort; NSCLC, non-small-cell lung cancer; MAF. minor allele frequency

Figure S2 OS of patients by *TSC1* genetic variant in the testing (A), validation (B), and combined (C) sets by Kaplan–Meier plot**.** MST, median survival time, months; CI, confidence interval

Supplementary Fig S1. Patients' enrollment and study scheme

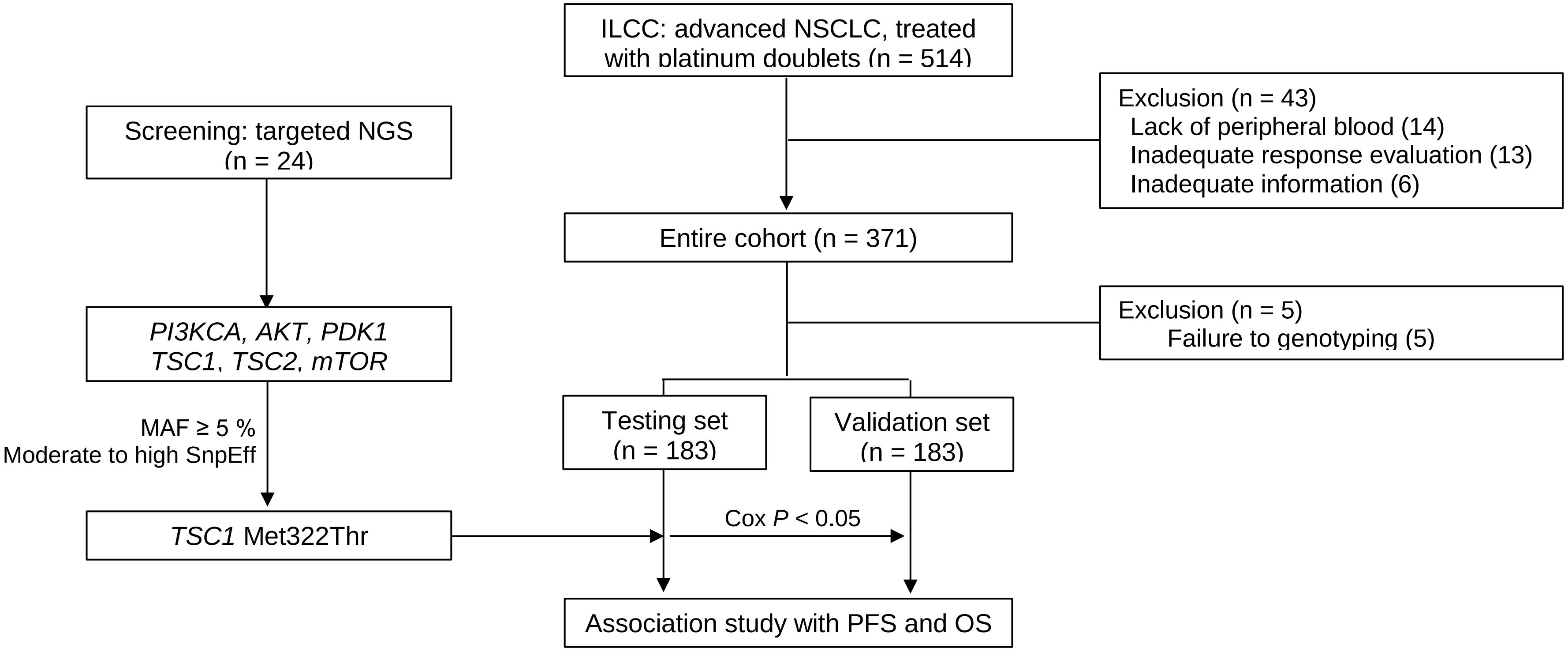

Abbreviations: ILCC, Inha Lung Cancer Cohort; NSCLC, non-small-cell lung cancer; MAF. minor allele frequency; PFS, progression-free survival; OS, overall survival

Supplementary Fig S2. Overall survival of the patients by genetic variation of *TSC1* gene in testing (A), validation (B), and combined (C) sets: Kaplan-Meier plot

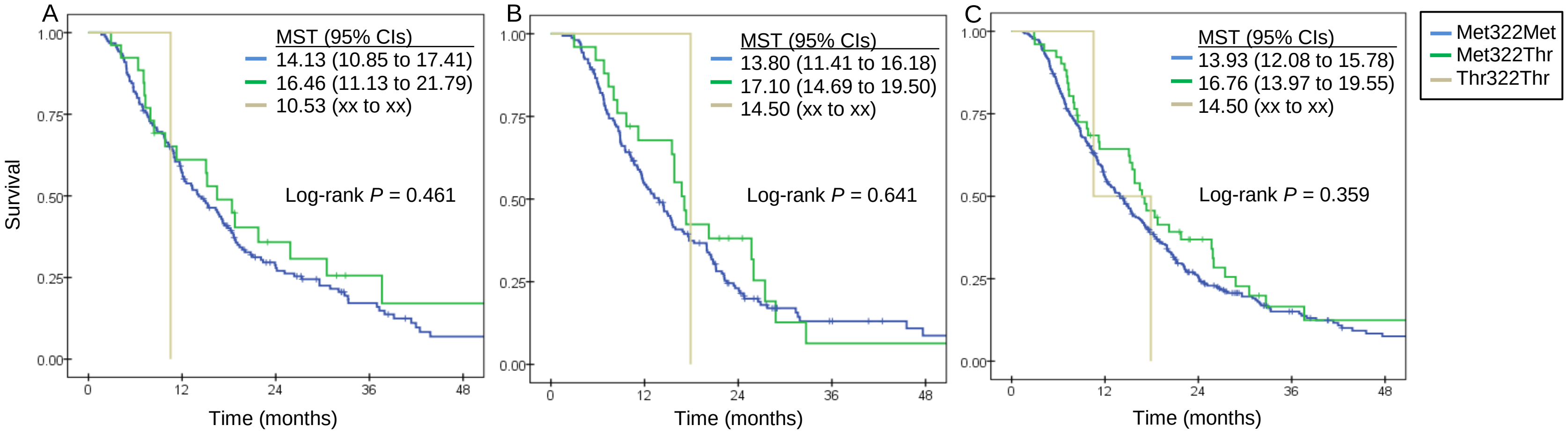

Abbreviations: MST, median survival time, months; CI, confidence interval

Supplementary Information

Content:

Table S1 Genetic variants of *TSC1* and *TSC2* identified by next-generation sequencing

Table S2 PFS of patients by clinical characteristics and *TSC1* gene variants in the testing, validation, and combined sets

Supplementary Table S1. Information on genetic variations of *TSC1* or *TSC2* gene and their related genes identified by next generation sequencing

GENE	Chromosome	Position	SNPID	Minor allele frequencies	Call rate	HWE Permutation test	Effect	Impact
	9	135786904	rs1073123	0.0833	1	1	non-synonymous	moderate
	9	135776612	rs77262230	0.0208	1	1	intron	low
	9	135773164	rs12350315	0.0833	1	1	intron	low
	9	135776341	rs7020438	0.0833	1	1	intron	low
	9	135776845	rs7020175	0.0833	1	1	intron	low
	9	135777121	rs11243931	0.0833	1	1	intron	low
	9	135788227	rs7875422	0.0833	1	1	intron	low
	9	135788272	rs7875558	0.0833	1	1	intron	low
	9	135788287	rs7865232	0.0833	1	1	intron	low
	9	135776925	rs75802666	0.25	1	0.5964	intron	low
	9	135776034	rs1076160	0.4583	1	0.6797	intron	low
	9	135781239	rs118203567	0.0625	1	1	synonymous	low
	9	135772717	rs4962081	0.0833	1	1	synonymous	low
	9	135782221	rs7862221	0.0833	1	1	synonymous	low
	9	135804139	rs80258442	0.0625	1	1	upstream	low
	9	135777793	chr9_135777793	0.0208	1	1	downstream	modifier
TSC1	9	135782769	rs118203508	0.0208	1	1	downstream	modifier
	9	135796621	chr9_135796621	0.0208	1	1	downstream	modifier
	9	135797655	chr9_135797655	0.0208	1	1	downstream	modifier
	9	135780223	chr9_135780223	0.0417	1	1	downstream	modifier
	9	135777451	rs7044064	0.0833	1	1	downstream	modifier
	9	135779530	rs12345576	0.0833	1	1	downstream	modifier
	9	135782277	rs7872606	0.0833	1	1	downstream	modifier
	9	135782479	rs7872860	0.0833	1	1	downstream	modifier
	9	135767185	rs11553763	0.125	1	1	UTR_3_prime	modifier
	9	135767488	chr9_135767488	0.0208	1	1	UTR_3_prime	modifier
	9	135767517	chr9_135767517	0.0208	1	1	UTR_3_prime	modifier
	9	135770816	chr9_135770816	0.0208	1	1	UTR_3_prime	modifier
	9	135771567	chr9_135771567	0.0208	1	1	UTR_3_prime	modifier
	9	135769688	rs10491534	0.0417	1	1	UTR_3_prime	modifier
	9	135767943	rs1050700	0.2292	1	0.2889	UTR_3_prime	modifier
	9	135770347	rs2809244	0.375	1	0.6666	UTR_3_prime	modifier
	9	135770300	rs2809243	0.4167	1	1	UTR_3_prime	modifier
TSC2	16	2129027	chr16_2129027	0.0208	1	1	non-synonymous	moderate
	16	2130243	rs45517295	0.0208	1	1	non-synonymous	moderate

	16	2111779	rs17135764	0.25	1	1	intron	low
	16	2103408	chr16_2103408	0.0208	1	1	synonymous	low
	16	2113009	chr16_2113009	0.0208	1	1	synonymous	low
	16	2097110	rs2516740	0.0208	1	1	upstream	low
	16	2097158	rs2516739	0.0208	1	1	upstream	low
	16	2105115	chr16_2105115	0.0208	1	1	upstream	low
	16	2108740	chr16_2108740	0.0208	1	1	upstream	low
	16	2112481	chr16_2112481	0.0208	1	1	upstream	low
	16	2114033	chr16_2114033	0.0208	1	1	upstream	low
	16	2123248	chr16_2123248	0.0208	1	1	upstream	low
	16	2124148	chr16_2124148	0.0208	1	1	upstream	low
	16	2107489	rs2072314	0.0417	1	1	upstream	low
	16	2107288	chr16_2107288	0.0625	1	1	upstream	low
	16	2111571	chr16_2111571	0.0625	1	1	upstream	low
	16	2110571	rs2074968	0.3125	1	1	upstream	low
	16	2120402	rs7187438	0.3125	1	0.6353	upstream	low
	16	2106475	chr16_2106475	0.0208	1	1	downstream	modifier
	16	2106534	chr16_2106534	0.0208	1	1	downstream	modifier
	16	2131993	rs117153104	0.0417	1	1	downstream	modifier
	16	2115819	rs8063461	0.2083	1	1	downstream	modifier
	3	178916519	rs11709323	0.0833	1	1	intron	low
	3	178918696	chr3_178918696	0.0208	1	1	downstream	modifier
	3	178921838	chr3_178921838	0.0625	1	1	downstream	modifier
	3	178927111	rs56143971	0.1042	1	1	intron	low
PIK3CA	3	178927149	rs55685804	0.1042	1	1	intron	low
	3	178927345	rs3729682	0.1042	1	1	intron	low
	3	178935799	rs1568205	0.1042	1	1	upstream	low
	3	178942176	rs17849074	0.0417	1	1	intron	low
	3	178943947	chr3_178943947	0.0208	1	1	downstream	modifier
	14	105235558	rs2498801	0.2708	1	1	downstream	modifier
	14	105235824	rs58565216	0.0208	1	1	UTR_3_PRIME	modifier
	14	105235825	rs17846828	0.0208	1	1	UTR_3_PRIME	modifier
	14	105235860	rs3803305	0.0208	1	1	UTR_3_PRIME	modifier
AKT1	14	105236287	rs35416681	0.0208	1	1	UTR_3_PRIME	modifier
	14	105236557	rs17846826	0.0208	1	1	UTR_3_PRIME	modifier
	14	105237401	rs7140735	0.0417	1	1	INTRON	low
	14	105238670	rs8192700	0.0208	1	1	INTRON	low
	14	105238954	rs61761200	0.0208	1	1	INTRON	low
	14	105239146	rs3803304	0.1042	1	1	INTRON	low

	14	105239192	rs2494732	0.1667	1	1	intron	low
	14	105239894	rs1130233	0.3750	1	0.1938	synonymous_coding	low
	14	105240111	rs3730362	0.0208	1	1	intron	low
	14	105240450	rs78470418	0.0208	1	1	intron	low
	14	105240784	rs2494733	0.2917	1	1	intron	low
	14	105240885	rs2494734	0.2708	1	1	intron	low
	14	105241576	rs3730344	0.0208	1	1	intron	low
	14	105241660	rs17846812	0.0208	1	1	intron	low
	14	105242228	rs2498797	0.2917	1	1	intron	low
	14	105242831	rs3001371	0.3125	1	1	intron	low
	14	105242926	rs17846822	0.0208	1	1	intron	low
	14	105242966	rs2494735	0.2708	1	1	intron	low
	14	105243435	rs2498795	0.4375	1	0.2415	intron	low
	14	105246325	rs2494737	0.2708	1	0.6357	upstream	low
	14	105246384	rs17846818	0.0208	1	1	upstream	low
	14	105246407	rs3730358	0.0625	1	1	upstream	low
	14	105246565	rs17846816	0.0208	1	1	upstream	low
	14	105246681	rs35635404	0.0208	1	1	upstream	low
	14	105246686	rs2494738	0.4583	1	0.6797	upstream	low
	14	105246692	rs12588965	0.0208	1	1	upstream	low
	14	105246989	rs2494739	0.4583	1	0.6797	upstream	low
	14	105259706	rs10138227	0.0833	1	1	UTR_5_prime	modifier
	14	105259734	rs1130214	0.0833	1	1	UTR_5_prime	modifier
	14	105260301	rs28634999	0.0208	1	1	UTR_5_prime	modifier
	14	105261123	rs117096287	0.0625	1	1	upstream	low
	14	105261419	rs10144641	0.0208	1	1	upstream	low
	14	105262368	rs2498786	0.1458	1	1	upstream	low
	14	105262718	chr14_105262718	0.0208	1	1	upstream	low
	14	105262781	rs74090038	0.0833	1	1	upstream	low
	14	105262912	rs2494750	0.3125	1	1	upstream	low
	14	105262961	rs2494751	0.3125	1	1	upstream	low
	14	105263143	chr14_105263143	0.0625	1	1	upstream	low
	14	105263227	rs117871152	0.0208	1	1	upstream	low
	14	105263608	rs2494752	0.2917	1	0.6211	upstream	low
	1	11166480	chr1_11166480	0.0208	1	1	downstream	modifier
	1	11166713	rs2536	0.2083	1	1	UTR_3_prime	modifier
mTOR	1	11167146	rs12139042	0.2083	1	1	stop_gained	high
	1	11167760	rs12117235	0.2083	1	1	downstream	modifier
	1	11167829	rs12117241	0.2083	1	1	downstream	modifier

1	11169676	rs2275525	0.2083	1	1	intron	low
1	11175176	rs2000393	0.1458	1	1	upstream	low
1	11175270	rs2275522	0.2083	1	1	upstream	low
1	11181147	rs17848567	0.0208	1	1	intron	low
1	11181327	rs11121691	0.0417	1	1	synonymous_coding	low
1	11181457	rs17235633	0.2083	1	1	intron	low
1	11181630	rs12143194	0.2083	1	1	intron	low
1	11184489	rs868080	0.2083	1	1	intron	low
1	11186897	rs3737611	0.2083	1	1	downstream	modifier
1	11187342	chr1_11187342	0.0208	1	1	downstream	modifier
1	11187477	chr1_11187477	0.0208	1	1	downstream	modifier
1	11187662	chr1_11187662	0.0208	1	1	downstream	modifier
1	11189191	rs3730379	0.2083	1	1	downstream	modifier
1	11190546	rs17848555	0.0208	1	1	downstream	modifier
1	11192956	rs2275526	0.2083	1	1	synonymous_coding	low
1	11194591	rs3730381	0.2083	1	1	upstream	low
1	11194667	chr1_11194667	0.0208	1	1	upstream	low
1	11194836	rs12142442	0.2083	1	1	upstream	low
1	11199149	rs2275528	0.2083	1	1	non_synonymous_coding	moderate
1	11205058	rs1057079	0.2500	1	1	non_synonymous_coding	moderate
1	11205340	rs74630019	0.0208	1	1	upstream	low
1	11206690	rs2275942	0.2083	1	1	upstream	low
1	11209899	rs17036411	0.2083	1	1	upstream	low
1	11217665	rs72871466	0.2083	1	1	intron	low
1	11264828	rs2273127	0.2083	1	1	intron	low
1	11269796	rs1010447	0.0833	1	1	intron	low
1	11272529	rs28730685	0.0208	1	1	non_synonymous_coding	moderate
1	11273418	rs12116957	0.2083	1	1	intron	low
1	11276053	chr1_11276053	0.0417	1	1	intron	low
1	11288758	rs1064261	0.0833	1	1	synonymous_coding	low
1	11298362	chr1_11298362	0.0208	1	1	intron	low
1	11301714	rs1135172	0.0833	1	1	synonymous_coding	low
1	11301841	rs2076656	0.2083	1	1	intron	low
1	11302065	rs11121706	0.0833	1	1	intron	low
1	11303153	rs12141961	0.2083	1	1	intron	low
1	11303383	rs28730693	0.0417	1	1	intron	low
1	11307412	rs78613694	0.1042	1	0.2057	intron	low
1	11308509	rs56797473	0.2083	1	1	intron	low
1	11317310	rs12121319	0.2083	1	1	intron	low

	1	11318236	rs7525957	0.0833	1	1	intron	low
	1	11318763	rs2076657	0.2917	1	0.6211	intron	low
	1	11318885	rs17848582	0.0625	1	1	intron	low
	1	11318983	rs12142905	0.2083	1	1	intron	low
	1	11322156	rs1883965	0.0833	1	1	intron	low
	1	11322565	rs2295079	0.3125	1	0.3567	UTR_5_prime	modifier
	1	11322620	rs17027474	0.0208	1	1	upstream	low
	1	11322628	rs2295080	0.2917	1	0.6211	upstream	low
	2	173418939	rs10178654	0.1250	1	0.298	upstream	low
	2	173418990	rs10202550	0.1250	1	0.298	upstream	low
	2	173419009	rs74807097	0.0208	1	1	upstream	low
	2	173419115	rs77953241	0.0208	1	1	upstream	low
	2	173419805	rs10181851	0.2500	1	0.1131	upstream	low
	2	173420609	rs114945843	0.0417	1	1	upstream	low
	2	173435757	rs13394924	0.1250	1	0.298	downstream	modifier
	2	173460169	chr2_173460169	0.0208	1	1	intron	low
	2	173460195	rs13392808	0.1250	1	0.298	intron	low
	2	173460336	rs79485922	0.0208	1	1	intron	low
PDK1	2	173460640	rs12693005	0.1250	1	0.298	synonymous_coding	low
	2	173460803	rs1530864	0.1250	1	0.298	UTR_3_prime	modifier
	2	173461090	rs1530865	0.1250	1	0.298	UTR_3_prime	modifier
	2	173461799	rs70937083	0.1250	1	0.298	downstream	modifier
	2	173461820	rs11904158	0.2500	1	0.1131	downstream	modifier
	2	173462230	rs11904366	0.1250	1	0.298	downstream	modifier
	2	173462593	rs70937084	0.1250	1	0.298	UTR_3_prime	modifier
	2	173463138	rs2357637	0.1042	1	0.2057	UTR_3_prime	modifier
	2	173463175	chr2_173463175	0.0208	1	1	UTR_3_prime	modifier
	2	173463646	rs70937086	0.0208	1	1	UTR_3_prime	modifier
	2	173464187	rs13388338	0.1250	1	0.298	downstream	modifier

Supplementary Table S2. Progression-free survival of the patients by clinical characteristics and genetic variation of *TSC1* gene in testing, validation, and combined sets

Variables		Testing			Validation			Combined		
		mPFS	95% CI	log-rank P	mPFS	95% CI	log-rank P	mPFS	95% CI	log-rank P
Gender	Women	4.83	4.181 to 5.485	0.761	4.43	2.763 to 6.104	0.031	4.70	4.227 to 5.173	0.173
	Men	4.66	4.168 to 5.165		5.30	4.653 to 5.947		5.00	4.588 to 5.412	
Smoking habit	Never	4.63	3.768 to 5.498	0.613	5.10	4.510 to 5.690	0.626	4.40	3.564 to 5.236	0.009
	Ever	4.93	4.407 to 5.460		4.16	2.816 to 5.517		5.10	4.763 to 5.437	
ECOG PS	0 to 1	4.90	4.327 to 5.473	0.303	5.10	4.510 to 5.690	0.626	5.00	4.620 to 5.380	0.351
	2 or more	4.56	3.992 to 5.142		4.16	2.816 to 5.517		4.43	3.775 to 5.091	
Histology	ADC	4.00	3.020 to 4.980	0.001	4.60	3.900 to 5.300	0.004	4.46	3.834 to 5.099	<0.001
	SQC	5.20	4.656 to 5.743		6.26	5.653 to 6.880		5.76	4.911 to 6.622	
	Others	5.80	3.380 to 8.220		4.86	2.468 to 7.265		4.86	3.041 to 6.692	
Stage	IIIA	5.33	2.608 to 8.059	0.056	7.10	4.259 to 9.941	<0.001	7.06	4.367 to 9.767	<0.001
	IIIB	5.20	4.422 to 6.978		6.00	5.036 to 6.964		5.00	5.020 to 6.180	
	IV	4.56	3.829 to 5.305		4.86	4.306 to 5.427		4.63	4.236 to 5.031	
First line regimens	Platinum Plus									0.002
	Gemcitabine	5.00	4.329 to 5.671	0.341	5.10	4.356 to 5.844	0.002	5.06	4.659 to 5.474	
	Taxane	4.30	3.139 to 5.461		4.70	3.817 to 5.583		4.60	3.938 to 5.262	
	Irinotecan	4.66	4.496 to 4.838		5.93	4.360 to 7.506		4.86	4.326 to 5.407	
	Pemetrexed	4.00	0.594 to 7.406		4.43	2.398 to 6.469		4.00	2.268 to 5.732	
	Others	9.16	4.515 to 13.819		26.90	x to x		10.40	6.796 to 14.004	
Response	CR or PR	5.76	4.405 to 7.128	<0.001	6.56	5.937 to 7.196	<0.001	6.33	5.666 to 7.000	<0.001
	SD	5.56	4.749 to 6.384		5.30	4.883 to 5.717		5.30	4.788 to 5.812	
	PD	2.26	1.803 to 2.731		2.33	2.114 to 2.553		2.30	2.081 to 2.519	
	Not evaluated	5.33	xx to xx		-	-		5.33	xx to xx	
TSC1 Met322Thr	Met/Met	4.63	4.186 to 5.081	<0.001	4.90	4.309 to 5.491	<0.001	4.70	4.356 to 5.044	<0.001
	Met/Thr	5.93	4.018 to 7.849		6.46	4.312 to 8.621		6.10	4.684 to 7.516	
	Thr/Thr	1.36	xx to xx		1.43	x to x		1.36	xx to xx	

Abbreviations: mPFS, median progression free survival, months; ECOG PS, Eastern Cooperative Oncology Group performance status; SQC, squamous cell carcinoma; ADC, adenocarcinoma; CR, complete remission; PR, partial remission; SD, stable disease; PD, progressive disease
